# The Berlin bimanual test for stroke survivors (BeBiT-S): evaluating exoskeleton-assisted bimanual motor function after stroke

**DOI:** 10.1186/s12984-025-01822-6

**Published:** 2025-12-08

**Authors:** Mareike Vermehren, Annalisa Colucci, Cornelius Angerhöfer, Niels Peekhaus, Won-Seok Kim, Won Kee Chang, Hyunji Kim, Volker Hömberg, Nam-Jong Paik, Surjo R. Soekadar

**Affiliations:** 1 Clinical Neurotechnology Laboratory, Dept. of Psychiatry and Neurosciences, Charité Campus Mitte (CCM), Charité – Universitätsmedizin Berlin, Charitéplatz 1, 10117 Berlin, Germany; 2 Department of Rehabilitation Medicine, Seoul National University College of Medicine, Seoul National University Bundang Hospital, 82, Gumi-Ro 173 Beon-Gil, Bundang-Gu, Seongnam-Si, Gyeonggi-Do 13620 Republic of Korea; 3 Department of Health Science and Technology, Graduate School of Convergence Science and Technology, Seoul National University KR, Seoul, South Korea; 4 SRH Gesundheitszentrum Bad Wimpfen GmbH, Bad Wimpfen, Germany

**Keywords:** Stroke, Exoskeleton, Bimanual task performance, Brain-computer interface (BCI), Clinical assessment, Assistive robotics

## Abstract

**Background:**

Brain/neural hand exoskeletons (B/NHEs) can restore motor function after severe stroke, enabling bimanual tasks critical for various activities of daily living. Yet, reliable clinical tests for assessing bimanual function compatible with B/NHEs are lacking*.* Here, we introduce the Berlin Bimanual Test for Stroke (BeBiT-S), a 10-task assessment focused on everyday bimanual activities, and evaluate its psychometric properties as well as compatibility with assistive technologies such as B/NHEs.

**Methods:**

BeBiT-S tasks were selected based on their relevance to daily activities, representation of various grasp types, and compatibility with current (neuro-)prosthetic devices. A scoring system was developed to assess key aspects of bimanual function—including reaching, grasping, stabilizing, manipulating, and lifting—based on video recordings of task performance. The BeBiT-S was administered without support of assistive technology (*unassisted condition)* to 24 stroke survivors (mean age = 56.5 years; 9 female) with upper-limb hemiparesis. We evaluated interrater reliability through the intraclass correlation coefficient (ICC) and construct validity through correlations with the Chedoke Arm and Hand Activity Inventory (CAHAI), and Stroke Impact Scale (SIS). A subgroup of 15 stroke survivors (mean age 50.3 years, 5 female) completed a second session supported by a B/NHE (B*/NHE-assisted condition)* to assess the BeBiT-S’ sensitivity to change related to B/NHE-application.

**Results:**

The BeBiT-S demonstrated high interrater reliability in both the unassisted (ICC = 0.985, *P* < .001) and B/NHE-assisted (ICC = 0.862, *P* < .001) conditions. Unassisted BeBiT-S scores correlated with the CAHAI-8 (r(22) = 0.95, *P* < .001) and the SIS subscales “strength” (r(20) = 0.53, *P* = .012) and “hand function” (r(20) = 0.50, *P* = .018), indicating construct validity. BeBiT-S scores improved significantly with B/NHE assistance (Mdn = 60, P < .05), compared to when no assistance was provided (Mdn = 38, P < .05), demonstrating the test’s sensitivity to change following the application of a B/NHE.

**Conclusions:**

The findings support that the BeBiT-S is a reliable and valid tool for evaluating bimanual task performance in stroke survivors and is compatible with the use of assistive technology such as B/NHEs.

*Trial registration* NCT04440709, submitted June 18th, 2020.

**Supplementary Information:**

The online version contains supplementary material available at 10.1186/s12984-025-01822-6.

## Introduction

Hemiparesis, characterized by weakness or partial paralysis on one side of the body, impacts over 70% of stroke survivors [1]. frequently leading to enduring disability and diminished quality of life. Thus, regaining upper-limb function is critical for increasing independence in daily life activities, and has been identified as a priority by stroke survivors, especially in long-term treatment [2].

In instances of severe paralysis accompanied by complete loss or very limited residual hand function, conventional treatment approaches like constrained induced movement therapy (CIMT) or standard occupational practices often cannot be applied as they rely on residual function [3]. To fill this gap in post-stroke rehabilitation, brain-computer interface (BCI)-driven exoskeletons emerged as a promising treatment option for restoring upper limb function in stroke survivors [4–7]. By converting neural activity into control signals of robotic orthoses, such devices enable functional, user-initiated movement execution even in case of complete loss or very limited residual hand function [8]. This way, BCI-based devices allow patients to use the affected limb during therapy sessions, potentially mitigating the phenomenon of “learned non-use” which refers to the tendency to avoid using the affected limb. Recent meta-analyses confirm that repeated use of BCI-driven robotic devices can promote functional and structural plasticity triggering motor recovery [6, 9, 10].

In recent years, technological advances resulted in the development of lightweight robotic actuators, portable and easy-to-use brain recording devices, and reliable control strategies [4].

This development has enabled the emergence of brain/neural hand exoskeletons (B/NHEs) -devices that integrate brain signals with other biosignals such as electrooculography (EOG) or electromyography (EMG)—which hold significant promise for clinical use beyond the laboratory setting [8, 11]. By leveraging multiple biosignals, including those related to eye movements, these systems enable robust and safe control paradigms, paving the way for their effective implementation as rehabilitative tools [4, 11–13].

Although B/NHEs have the potential to support motor recovery in both unimanual and bimanual contexts, targeting bimanual function is particularly relevant in stroke rehabilitation [14]. Patients with hemiplegia often compensate for unilateral deficits during one-handed tasks; however, bimanual activities more accurately reflect real-world functional demands and may foster greater engagement and motivation during therapy. To assess improvements in bimanual function and validate emerging neuroprosthetic-based rehabilitative protocols, appropriate evaluation tools are needed [15]. Such evaluation tools are also essential to establish precision rehabilitation frameworks that use advanced computational methods, including collaborative AI, leveraging diverse datasets and data sources to create individualized digital profiles [16]. 

However, no standardized clinical tests currently exist to evaluate performance in bimanual activities of daily living (ADL) during B/NHE-assisted tasks in stroke survivors. Such a test should be designed to accommodate the use of B/NHEs while thoroughly monitoring subtle changes in upper limb functionality. Moreover, it must reflect changes in patients’ capability to perform ADLs affected by stroke, which usually requires both upper limbs to act together in a highly coordinated and efficient way [17]. Thus, arm rehabilitation strategies targeting only the weaker arm are limited in their effectiveness, suggesting to shift clinical rehabilitation towards bimanual training [14]. Current clinical assessments lack such necessary features since they typically emphasize unilateral, repetitive movements (such as the Fugl-Meyer Assessment for upper extremity, FMA-UE) or unilateral functional tasks (like the Action Research Arm Test, ARAT, Toronto Rehabilitation Institute Hand Function Test, TRI-HFT, or Wolf Motor Function Test, WMFT). The Assisting Hand Assessment (AHA), a standardized tool that evaluates how effectively the impaired hand is used during real-world bimanual activities, partially addresses this gap; however, it is primarily designed for children and centers on play-based tasks, limiting its applicability to adult stroke populations. Others, such as the Box and Block Test or Jebsen Taylor Hand Function Test (JTHFT), are based on timing-based metrics, making them less suitable for use in conjunction with assistive exoskeletons, which may alter movement speed or task execution and thus limit score comparability and interpretability.

Currently, there is only one performance-based assessment tool that evaluates bimanual hand function in ADLs after stroke, the Chedoke Arm and Hand Activity Inventory (CAHAI) [18] . While the CAHAI offers valuable insights into functional bimanual tasks that are important in stroke patients’ daily life, it is less suited for evaluating task performance in the context of assistive technologies such as B/NHEs, as certain tasks are incompatible with the use of B/NHEs (e.g., wringing out a wet washcloth). Additionally, the evaluation of tasks in the CAHAI focuses exclusively on the degree of independence, which is inherently biased in the use of assistive devices.

An initial effort to address this gap in clinical assessments—by developing tests that are both representative of everyday bimanual activities and compatible with current neuroprosthetic technologies—was made by Angerhöfer et al., who introduced the Berlin Bimanual Test for Tetraplegia (BeBiT-T) [19]. The novel assessment tool evaluates bimanual task performance in daily life activities for tetraplegic individuals. Given the marked distinctions in motor symptoms between tetraplegia and stroke – including spasticity, residual motor function, trunk control, and weakness or partial paralysis on both sides of the body – there is a critical need to establish a similar test for stroke survivors. Here, we introduce such test which was inspired by the BeBiT-T but is specifically tailored for stroke survivors, who typically present with unilateral rather than bimanual motor impairment. Comprising 10 bimanual tasks directly relevant to the daily life of stroke survivors, the Berlin Bimanual Test for Stroke (BeBiT-S) is designed to seamlessly integrate with the use of modern technologies such as B/NHEs, thereby complementing existing ADL assessments such as the CAHAI.

In the following, we present the rationale behind the selection of items for the BeBiT-S and evaluate the tests’ reliability and validity. The primary objective of this study is to validate the BeBiT-S as a clinical assessment tool for evaluating bimanual function in stroke survivors. Given that the key innovation of the BeBiT-S lies in its compatibility with assistive technologies—particularly B/NHEs—demonstrating this compatibility is a critical component of the validation process. Thus, as a secondary outcome, we examined the test’s sensitivity to detect change by comparing performance with and without B/NHE-assistance.

## Methods

### Development of the Berlin bimanual test for stroke (BeBiT-S)

**Fig. 1:**
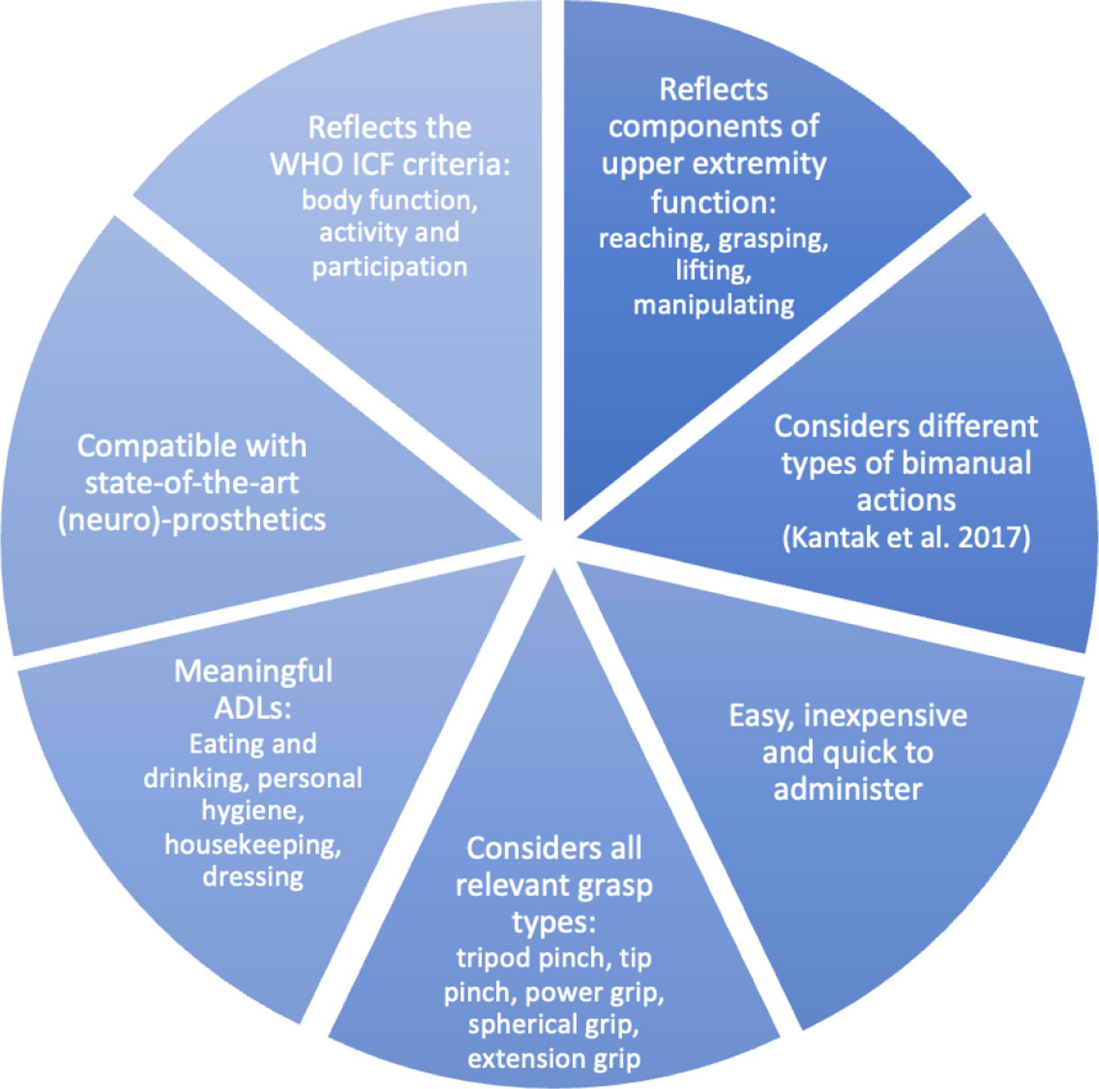
Theoretical background: Essential requirements for the selection of tasks

The development of the BeBiT-S involved a systematic three-stage process. In phase I, a theoretical framework was established (Fig. 1), and a pool of potential test items was generated by an interdisciplinary research team including experts in classical test theory, physicians, medical engineers, psychologists, and physical therapists. In phase II, 10 test items were selected based on the theoretical framework. Our criteria included coverage of various categories of ADLs, such as eating and drinking, housekeeping, dressing, and personal hygiene. Additionally, we ensured representation of all relevant grasp types essential for daily life, encompassing tripod pinch, tip pinch, power grip, spherical grip, and extension grip. In this way, the BeBiT-S aligns with the World Health Organization’s (WHO) criteria for the International Classification of Functioning, Disability and Health (ICF), considering body function and activity [20]. Items were further selected to represent diverse bimanual actions that are affected post-stroke, following the taxonomy of Kantak, Jax [14]. They distinguish between symmetric bimanual actions involving the use of homologous muscles, and asymmetric movements involving non-homologous muscles. Moreover, actions are distinguished in which each hand pursues an independent goal or both hands work together to accomplish a common task. In addition to these considerations, items were selected for easy and quick administration as well as inexpensiveness. In phase III, we screened the selected tasks regarding compatibility with assistive technologies such as B/NHEs, ensuring that the objects used could be effectively grasped and manipulated by the exoskeleton.

The refined BeBiT-S, as presented in its final version, comprises ten bimanual tasks specifically tailored to address the needs of stroke survivors in their daily lives. The materials required for the test are readily available in most clinical and research settings around the world, making it accessible on a global scale (Table 1).

**Table 1:** Overview of tasks and materials included in the Berlin Bimanual Test for Stroke

Task	Material	ADL category	Bimanual action
Open jar	Jar with screw lid, around 200 g	Eating and drinking	Asymmetric, common goal, cooperative
Open plastic food package	Zip lock bag filled with weight simulating a packet of crisps	Eating and drinking	Symmetric, common goal, cooperative
Open water bottle	Plastic water bottle (filled), screwed on by hand	Eating and drinking	Asymmetric, common goal, cooperative
Pour glass of water	Plastic water bottle (filled), water glass (empty)	Eating and drinking	Asymmetric, common goal, parallel
Eat with knife and fork	Medium resistance putty resembling the consistency of a piece of meat, table knife, fork	Eating and drinking	Asymmetric, common goal, cooperative
Clean a plate	Dinner plate, kitchen sponge	Housekeeping	Asymmetric, common goal, cooperative
Lift pot	Saucepan with flat handles	Housekeeping	Symmetric, common goal, cooperative
Unscrew toothpaste	Toothpaste with screw lid, > 50% full	Personal hygiene	Asymmetric, common goal, cooperative
Apply toothpaste on toothbrush	Toothpaste, toothbrush, and grip-easy rubber handles	Personal hygiene	Asymmetric, common goal, parallel
Close zipper of a jacket	Jacket or vest with zipper	Dressing	Asymmetric, common goal, cooperative

The tasks were selected to represent diverse bimanual actions based on the taxonomy proposed by Kantak, Jax [14]

The development of the BeBiT-S also involved the creation of a comprehensive scoring system to assess the primary components of bimanual function in ADLs, encompassing reaching, grasping, stabilizing, manipulating, and lifting. Scoring for the BeBiT-S relies on video recordings capturing participants’ performance of the designated tasks. An exemplary scoring sheet is provided in the appendix.

Each task is assigned a score ranging from 0 to 10 points, contributing to a maximum achievable score of 100. 20 points can be obtained for each *reaching* and *grasping* components, 10 points for *stabilizing*, 33 for *manipulating*, and 17 for *lifting*. In case of *reaching* and *grasping*, the maximum score is penalized if compensatory strategies are detected, such as hand-over-hand technique to reach an object or utilizing passive support of spasticity to hold an object. The scoring system categorizes object *manipulation* into three levels (no difficulties, slight difficulties, or great difficulties) while also considering the application of compensatory strategies, such as using both hands to manipulate a single object. No points are given if only the unaffected hand is used. Depending on the task, three or five points can be obtained for correctly *manipulating* an object. If applicable, two points are given for performing the task in a lifted position, when objects have no contact with any surface, including the patient’s trunk. One point is given for *stabilization* if the affected arm maintains a firm grip throughout the task. The BeBiT-S scoring system imposes no time constraint or limit to the number of attempts. The attempt ends when either the participant or the instructor believes that no better performance can be achieved. The best attempt is scored.

### Participants

We recruited 24 individuals with subacute or chronic post-stroke upper-limb hemiparesis (mean age 56.5 ± 16.8 years, 9 female). The mean age of female participants was 62.1 ± 12.3 years, while that of male participants was 53.1 ± 18.6 years. Recruitment and assessment of subacute stroke survivors (n = 9, < 6 months post-stroke) took place at SRH Gesundheitszentrum Bad Wimpfen, Germany. Chronic stroke survivors (n = 15, > 6 months post stroke) were assessed at Charité – Universitätsmedizin Berlin, P.A.N. Zentrum Berlin-Frohnau, or at their homes. Participant characteristics are depicted in Table 2 and 3. To be eligible for the study, potential participants had to fulfill the following requirements: stroke with upper-limb hemiparesis, age ranging from 18 to 85 years, cognitive ability to understand and follow instructions, and absence of any alcohol or drug addiction, terminal medical illness, or neurological or psychiatric condition other than stroke. The study was approved by the local ethics committee at Charité – Universitätsmedizin Berlin under number EA1/106/20. All participants provided written informed consent before enrollment.

**Table 2:** Participant characteristics

Participant	Age	Gender	Time post stroke (months)	Chronicity	Stroke type	Hemiparesis	Location	CAHAI-8 Score^a^	BeBiT-S Score unassisted	BeBiT-S Score B/NHE assisted^b^
01	48	m	44	chronic	n.a	right	cortical/mixed	18	31	47
02	42	m	1	subacute	hemorrhagic	left	subcortical	47	96	96
03	40	m	48	chronic	ischemic	left	subcortical	19.43	60	77
04	76	m	3	subacute	hemorrhagic	left	subcortical	14	31	44
05	62	f	11	chronic	ischemic	left	cortical/mixed	9	6	–
06	71	m	2	subacute	ischemic	left	Cortical/mixed	8	4	–
07	60	m	3	subacute	ischemic	right	subcortical	47	87	78
08	84	m	14	chronic	hemorrhagic	left	subcortical	13	29	–
09	43	f	27	chronic	ischemic	right	subcortical	12	26	59
10	83	f	2	subacute	ischemic	right	subcortical	43	90	–
11	61	m	4	subacute	ischemic	right	cortical/mixed	9.33	16	–
12	41	m	3	subacute	ischemic	right	cortical/mixed	18	69	–
13	70	f	3	subacute	ischemic	right	cortical/mixed	49.33	98	–
14	73	f	2	subacute	ischemic	left	cortical/mixed	10	10	–
15	31	m	16	chronic	ischemic	right	cortical/mixed	13	38	51
16	55	m	96	chronic	ischemic	right	cortical/mixed	12	18	–
17	74	m	144	chronic	ischemic	left	n.a	11	22	78
18	54	f	90	chronic	hemorrhagic	left	cortical/mixed	43	84	89
19	18	m	216	chronic	n.a	right	n.a	46	95	74
20	57	f	324	chronic	ischemic	right	subcortical	20	43	60
21	51	f	114	chronic	hemorrhagic	right	subcortical	8	4	51
22	66	f	96	chronic	ischemic	right	cortical/mixed	12	32	44
23	58	m	58	chronic	ischemic	right	cortical/mixed	32	66	70
24	37	m	7	chronic	hemorrhagic	left	cortical/mixed	17	27	51

m = male; f = female; B/NHE = Brain/Neural Hand Exoskeleton; CAHAI = Chedoke Arm and Hand Activity Inventory, BeBiT-S = Berlin Bimanual Test for Stroke

^a^otal score of the CAHAI-8 is 56, the higher the score the more assistance is necessary to complete the task

^b^15 participants performed the BeBiT-S unassisted and with support of a brain/neural hand exoskeleton (B/NHE-assisted). 9 participants performed the BeBiT-S unassisted only

**Table 3:** Demographic and clinical characteristics of participants by condition

Characteristic	Unassisted condition (n = 24)	B/NHE-assisted condition (n = 15)
Age, mean ± SD (range), years	56.5 ± 16.8 (18–84)	50.3 ± 15.8 (18–76)
Gender, n (%)		
Male	15 (62.5%)	10 (66.7%)
Female	9 (37.5%)	5 (33.3%)
Time since stroke, mean ± SD (range), months	55.3 ± 80.1 (1–324)	79.4 ± 91.3 (1–324)
Stroke type, n (%)		
Ischemic	16 (66.7%)	8 (53.3%)
Hemorrhagic	6 (25.0%)	5 (33.3%)
Not available	2 (8.3%)	2 (13.3%)
Affected hemisphere, n (%)		
Left	14 (58.3%)	9 (60.0%)
Right	10 (41.7%)	6 (40.0%)
Lesion location, n (%)		
Cortical	13 (54.2%)	6 (40.0%)
Subcortical	9 (37.5%)	7 (46.7%)
Not available	2 (8.3%)	2 (13.3%)
CAHAI-8 score, mean ± SD	21.6 ± 14.5	24.3 ± 15.2
BeBiT-S score unassisted, mean ± SD	45.1 ± 32.5	49.5 ± 29.6
BeBiT-S score B/NHE-assisted, mean ± SD	N/A	64.6 ± 16.9

*B/NHE* Brain/Neural Hand Exoskeleton, *CAHAI* Chedoke Arm and Hand Activity Inventory, *BeBiT-S* Berlin Bimanual Test for Stroke, *SD* standard deviation

### Study design

Participants performed the BeBiT-S twice—once without technological assistance (*unassisted condition*) and once supported by a brain/neural-controlled exoskeleton (*B/NHE-assisted condition*)—in a within-subject study design. These two conditions, referred to as *unassisted* vs. *B/NHE-assisted* from here on, were administered across two sessions, scheduled either on the same day or on separate days, depending on participant endurance and logistical constraints. The order of conditions was randomized, with 8 out of 15 participants completing the unassisted condition first.

All 24 participants completed the BeBiT-S unassisted. A subgroup of 15 participants (5 female, 10 male, 12 chronic, mean age 50.3 ± 15.8 years) also completed the B/NHE-assisted condition. The remaining 9 participants were unable to do so for the following reasons: (1) inability to follow the B/NHE control paradigm (n = 3), (2) inability to perform horizontal eye movements (n = 1), (3) exhaustion (n = 1), (4) technical issues (n = 1), (5) exoskeleton did not fit (n = 1), (6) time constraints (n = 2).

To assess the validity of the BeBiT-S, participants also completed the CAHAI-8[21]. The tasks included in the CAHAI-8 are as follows: (1) open a jar of coffee, (2) dial 911, (3) draw a line with a ruler, (4) pour a glass of water, (5) wring out a washcloth, (6) do up five buttons, (7) dry back with a towel, and (8) put toothpaste on a toothbrush. The CAHAI-8 was completed without B/NHE assistance. Finally, participants were asked to complete a subset of 4 domains of the Stroke Impact Scale (SIS) version 3.0 questionnaire [22]. The four selected domains included strength, ADLs, hand function, and percentage of recovery.

**Fig. 2:**
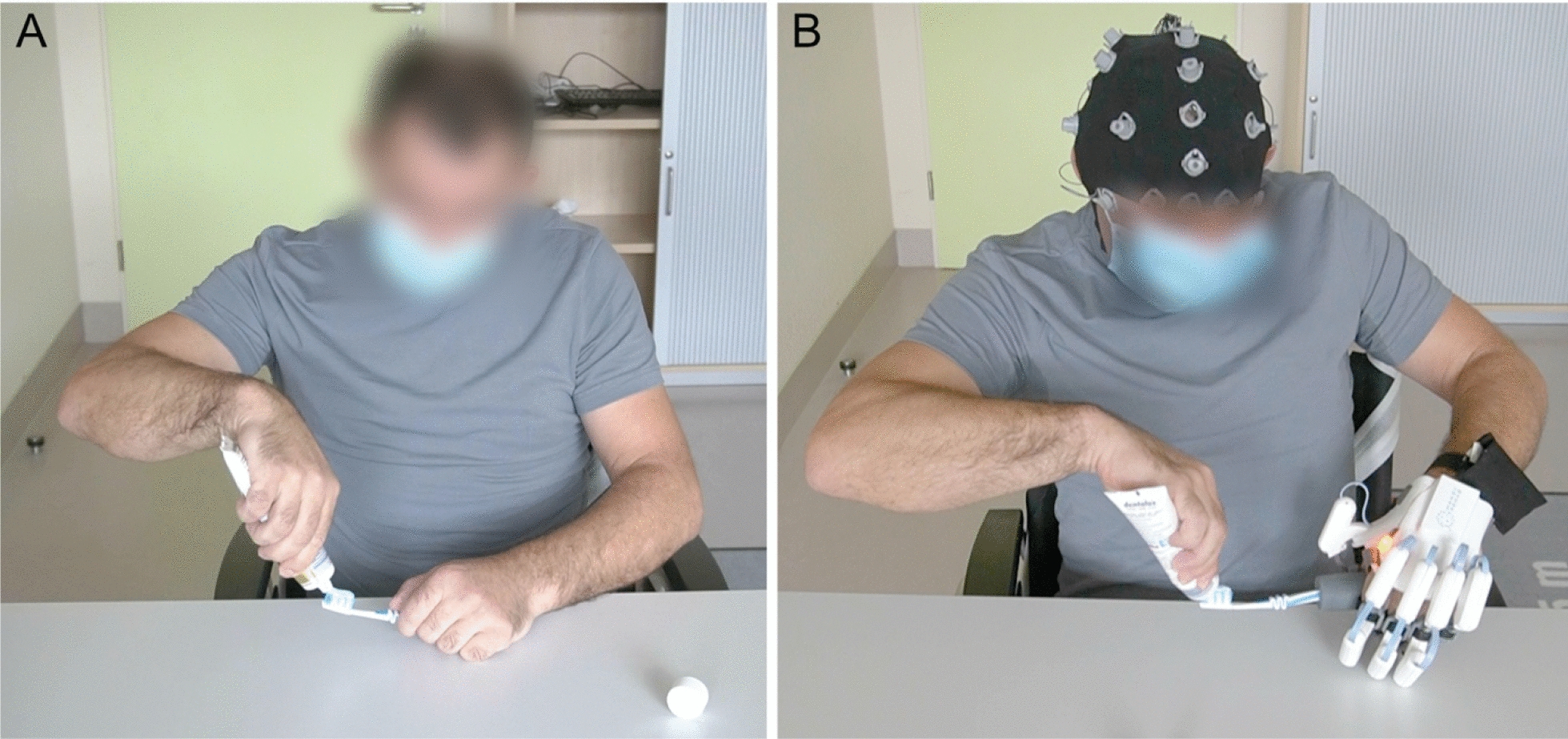
Experimental setup showing a participant performing the task *“apply toothpaste to toothbrush”* during the unassisted condition (**A**) and during the B/NHE-assisted condition (**B**), in which the participant is supported by a hand exoskeleton controlled through neural activity

In the B/NHE-assisted condition, the BeBiT-S was repeated in the same way as in the unassisted condition with participants operating a B/NHE to support grasping with their paretic hand (Fig. 2). Participants´ performance in both the unassisted and B/NHE-assisted condition was evaluated with the corresponding scoring sheet (Appendix). Ratings from three independent raters, all experienced in evaluating stroke survivors, were obtained to assess interrater reliability.

### Experimental setup

Participants were seated comfortably in a chair or wheelchair in front of a desk. Each BeBiT-S task was explained and demonstrated by the instructor. Participants were asked to perform the tasks as closely as possible to the instructor’s demonstration and were reminded of the importance of using both hands and avoiding compensatory strategies whenever possible. Each session was videotaped for scoring with a clear view of the materials throughout the task.

**Fig. 3:**
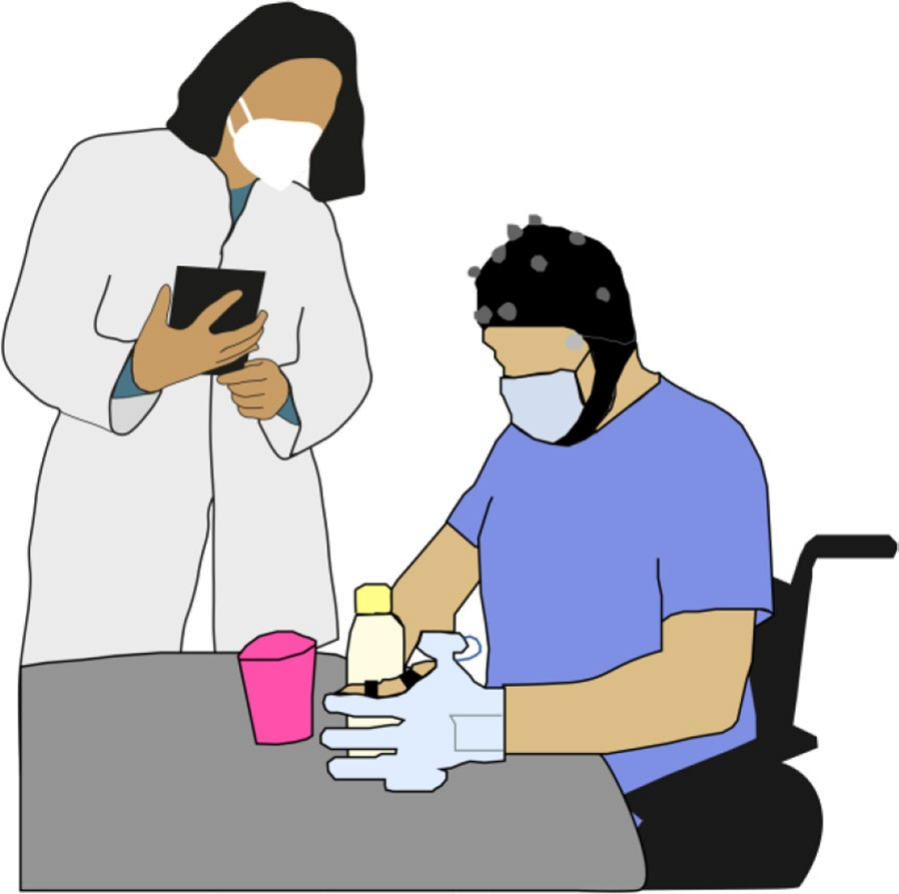
Experimental setup. The participant performs the BeBiT-S with the support of a hand exoskeleton on the paretic left hand. A hybrid brain-computer interface based on electroencephalography (EEG) and electrooculography (EOG) allows the participant to operate the hand exoskeleton

For the B/NHE-assisted condition, a wearable hand exoskeleton allowing firm grasping was attached to the participants paretic hand (Fig. 3). A hybrid brain-computer interface based on electroencephalography (EEG) and electrooculography (EOG) signals allowed the participant to operate the exoskeleton (HandyRehab by Zunosaki Ltd., Hong Kong). Detection of sensori-motor rhythm (8–13 Hz) event-related desynchronization (SMR-ERD) was translated to exoskeleton closing movements, while horizontal eye movements (horizontal oculoversion, HOV) were used to control exoskeleton opening movements and to stop unintentional closing. EEG was recorded from five conventional sites according to the international 10/20 system: F3, T3, C3, P3, and Cz for right-hand control, or F4, T4, C4, P4, and Cz for left-hand control of the B/NHE. Additionally, two electrodes were placed on the right and left outer canthus to record EOG. EEG and EOG signals were processed in real time through a custom BCI software (BeamBCI) based on the Python programming language. Signals were recorded at a sampling frequency of 250 Hz and bandpass filtered between 1 and 30 Hz. Power spectral density in the mu-range was estimated using Burg’s method [23]. A surface Laplacian filter was applied to increase signal-to-noise ratio at the target electrodes C3 or C4 [8]. During a short calibration session before the beginning of the measurement, participants learned to perform motor imagery or motor attempts to close the B/NHE, and maximum HOV to open the B/NHE. During the calibration, participants trained by means of visual cues on a computer screen. After the calibration, while performing the BeBiT-S tasks with the B/NHE, participants could decide when to open and close the exoskeleton in an uncued (asynchronous) paradigm.

### Data analysis

We assessed internal consistency and scale reliability for the unassisted BeBiT-S scores of the 10 tasks by calculating Cronbach’s Alpha and corrected item-total correlations. These analyses were performed using SPSS (v. 27). Following the guideline proposed by Kline [24], tasks with item-total correlation below r = 0.3 were discarded.

To quantify the level of agreement among the three raters in evaluating the BeBiT-S score, we computed the intraclass correlation coefficient (ICC) using SPSS (v. 27). The ICC was calculated separately for both conditions (unassisted and B/NHE-assisted) in a Two-Way Mixed model with absolute agreement.

To evaluate construct validity, we obtained CAHAI-8 scores for all participants and calculated the linear relationship between the unassisted BeBiT-S scores and the CAHAI-8 scores using Pearson’s correlation coefficient. Given that the CAHAI-8 is a performance-based assessment tool for evaluating bimanual hand function in post-stroke ADLs, it seemed most appropriate for assessing construct validity of the BeBiT-S. Furthermore, we computed the linear relationship of the unassisted BeBiT-S scores and four domains of the Stroke Impact Scale (strength, activities of daily living, hand function, percentage of recovery) by computing Pearson’s correlation coefficient.

To evaluate the BeBiT-S’ sensitivity to assess the impact of B/NHE use, we compared BeBiT-S scores with and without B/NHE-assistance with a Wilcoxon signed rank test. Moreover, we computed five Wilcoxon signed rank tests to assess changes within each component of hand function (reaching, grasping, stabilizing, manipulating, lifting).

To evaluate potential bias in the subgroup that completed the BeBiT-S with B/NHE-assistance (i.e., whether they were less impaired and demonstrate higher bimanual function unassisted), we computed a Mann–Whitney test comparing unassisted BeBiT-S scores between participants who completed both conditions and those who only completed the unassisted condition.

Prior to selecting non-parametric methods, data distributions were assessed using the Shapiro–Wilk test for normality. The significance level was set at *P* < 0.05.

## Results

### Validity and reliability

The Cronbach’s alpha coefficient calculated for the 10 tasks within the unassisted condition yielded an α of 0.97. All tasks demonstrated item-total correlations of r ≥ 0.82, surpassing the predefined cut-off level of r > 0.3 (Table 4).

**Table 4:** Internal consistency analysis

Tasks	Corrected item-total correlation	Cronbach's alpha if task deleted
Open jar	0.877	0.967
Open plastic food package	0.833	0.968
Open water bottle	0.832	0.968
Pour glass of water	0.936	0.964
Eat with knife and fork	0.820	0.969
Clean a plate	0.835	0.968
Lift a pot	0.867	0.967
Unscrew toothpaste	0.888	0.966
Apply toothpaste on toothbrush	0.867	0.967
Close zipper of a jacket	0.873	0.967

Interrater reliability in the unassisted condition yielded an ICC for single measures of 0.985 (95% confidence interval [CI] 0.969 to 0.993, *P* < 0.001). In the B/NHE-assisted condition the ICC for single measures was 0.862 (95% CI 0.432 to 0.962, *P* < 0.001).

**Fig. 4:**
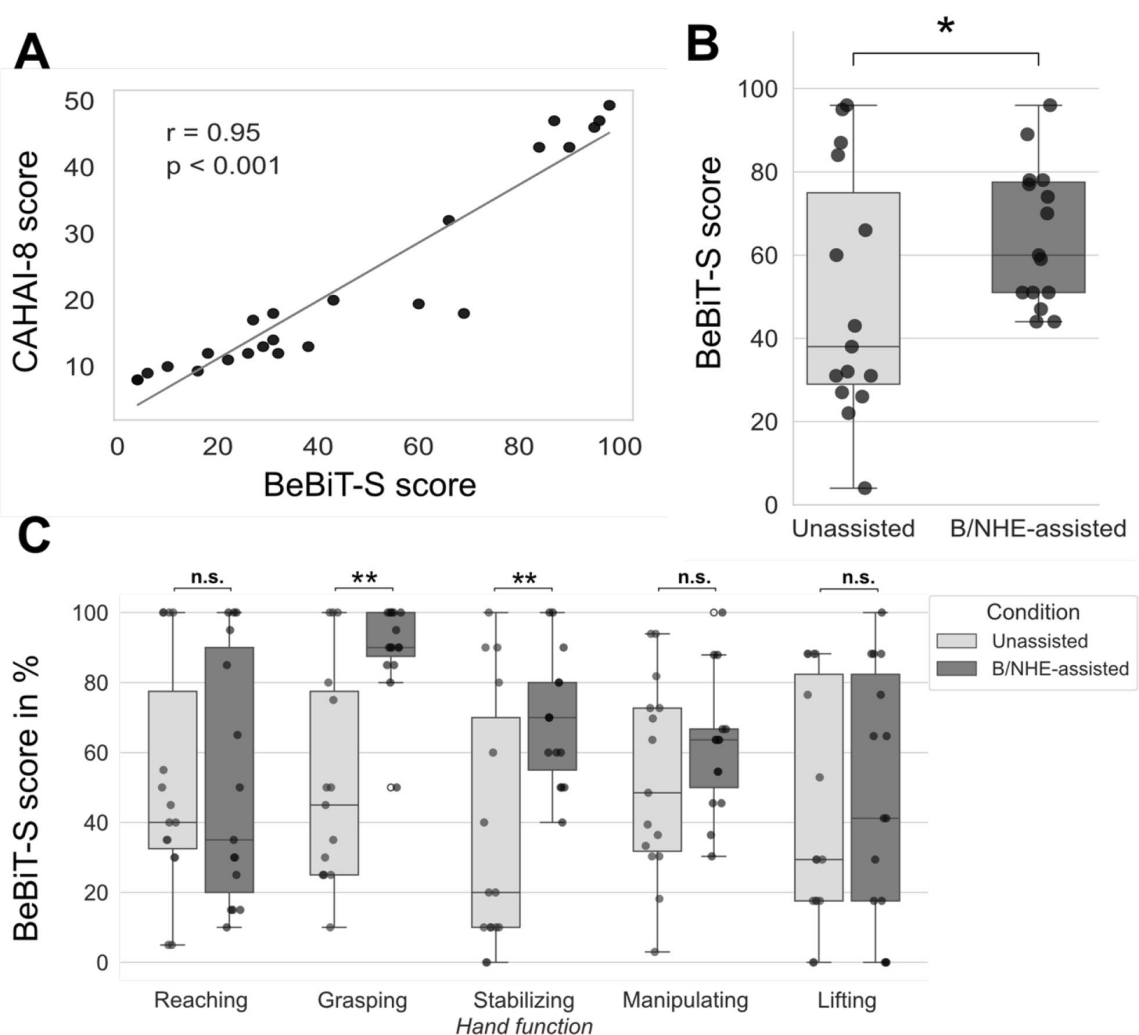
(**A**) Correlation between Berlin Bimanual Test for Stroke (BeBiT-S) and CAHAI-8, r(22) = 0.95, *P* < .001. (**B**) BeBiT-S scores in 15 participants performing the test without assistive technology and with a brain/neural hand exoskeleton (B/NHE). **A** Wilcoxon Signed Rank test showed a significant increase in scores with B/NHE-assistance (*P* < .05, r = 0.45). (**C**) Differences in BeBiT-S component scores for reaching, grasping, stabilizing, manipulating and lifting. Wilcoxon Signed Rank tests indicate that scores for grasping and stabilizing components improved significantly when using a B/NHE. * *P* < .05, ** *P* < .01, *** *P* < .001

A positive correlation was identified between unassisted BeBiT-S scores (M = 48.08, SD = 32.45) and CAHAI-8 scores (M = 22.13, SD = 14.94), r(22) = 0.95, *P* < 0.001, as depicted in Fig. 4A. The unassisted BeBiT-S scores correlated significantly with the scores of the SIS-1 domain “strength” (r(20) = 0.53, *P* = 0.012) and SIS-7 domain “hand function” (r(20) = 0.50, *P* = 0.018), but not with domains “ADLs” (r(20) = 0.245, *P* = 0.273) and “percentage of recovery” (r(18) = 0.27, *P* = 0.911).

The result of the Mann–Whitney test indicated no significant difference of the unassisted BeBiT-S scores between participants who completed both conditions (Mdn = 38) and those who completed only the unassisted condition (Mdn = 18, U = 47.5, *P* = 0.245).

### Comparison of unassisted and B/NHE-assisted conditions

A Wilcoxon Signed Rank test revealed an increase in BeBiT-S score from the unassisted (Mdn = 38) to the B/NHE-assisted (Mdn = 60) condition, z = 2.48, *P* = 0.013, with a medium effect size (r = 0.45) (Fig. 4B). This result underlines the sensitivity of the test in detecting changes in B/NHE applications.

Analysis of individual components using Wilcoxon Signed Rank tests indicate significant improvements in grasping (z = 3.11, *P* < 0.01) and stabilizing (z = 2.55, *P* < 0.01) during B/NHE-assistance (Fig. 4C). Non-significant improvements were observed in the manipulating (z = 1.64, *P* = 0.102) and lifting (z = 0.5, *P* = 0.619) components of the BeBiT-S score. The reaching component displayed a marginal decline, although not statistically significant (z = 1.25, *P* = 0.211).

## Discussion

The lack of reliable clinical tests for assessing bimanual hand function after stroke that are compatible with assistive technologies such as B/NHEs limits research into their clinical efficacy. Here, we addressed this critical gap and showed that the BeBiT-S is effective, reliable, and valid to assess bimanual motor function after stroke with and without B/NHEs. The central advantage of the BeBiT-S lies in its compatibility with state-of-the-art assistive technology, particularly B/NHEs, and its sensitivity to B/NHE-related motor improvements.

The BeBiT-S comprises ten bimanual tasks selected to be inexpensive as well as quick to administer and easy to implement in clinical settings. Tasks were chosen to represent bimanual actions affected after stroke, to be relevant to everyday life, to cover relevant grasp types, and finally, to be compatible with state-of-the-art (neuro-)prosthetic devices. Excellent internal consistency of the test was indicated by Cronbach’s alpha (0.97), exceeding the threshold for clinical instruments (> 0.9) as suggested by Nunally [25]. All ten tasks met the predefined cut-off level of item-total correlations of r > 0.3.

The high correlation coefficient (ICC) values, exceeding 0.85 with and without B/NHE-assistance, indicate consistent and reliable scoring across different raters. Raters were only given brief instructions included on the scoring sheet (see Appendix). This underlines the robustness of the BeBiT-S and feasibility for inexperienced personnel to apply it.

The highly positive correlation between the unassisted BeBiT-S scores and CAHAI-8 scores supports the BeBiT-S’ construct validity, indicating it effectively measures bimanual function in stroke survivors. Moreover, the unassisted BeBiT-S score also correlated with the Stroke Impact Scale (SIS) domains “strength” and “hand function”, providing additional support for the construct validity of the BeBiT-S. It is noteworthy, however, that the BeBiT-S did not correlate with SIS domains “ADLs” and “percentage of recovery” as originally anticipated. One reason for this could be the heterogeneity in chronicity in our participant group (from less than 3 months post-stroke to 27 years post-stroke), where the subjective nature of “percentage of recovery” becomes particularly apparent.

In our study, the condition with B/NHE-assistance was completed by a subgroup of 15 out of 24 total participants. To rule out a potential bias, we compared the unassisted BeBiT-S scores of participants who completed both conditions with those who only completed the unassisted condition. The absence of a difference suggests that lower bimanual function does not prevent B/NHE use. Looking more closely into the drop-out reasons for B/NHE use, two main reasons could be distinguished: *technical* and *non-technical* reasons*.* While technical reasons include issues related to the fit of the hand-exoskeleton, system failure or time constraints on the patient’s side, non-technical reasons include participant exhaustion, low motivation, or inability to follow the B/NHE control paradigm. Notably, those excluded for non-technical reasons were all within their first months after stroke (n = 5, on average 4.2 months after stroke), whereas technical reasons were more prevalent among chronic stroke survivors (3 out of 4 chronic). A notable observation was that controlling a B/NHE appeared more challenging for subacute stroke survivors compared to those in the chronic phase. This implies that incorporating B/NHE-enabled rehabilitation into sub-acute stroke cases might pose greater difficulty compared to chronic stroke scenarios. It underscores the importance of tailoring rehabilitation approaches to the specific needs and recovery stages of each individual [26, 27] Further research is needed to refine rehabilitation protocols to enhance their effectiveness and improve patient adherence.

The second main goal of our study was to demonstrate the compatibility of the BeBiT-S with assistive technologies such as B/NHEs. This is particularly important, given the lack of such tests that are, however, necessary to evaluate efficacy of B/NHE-related rehabilitation protocols. Our investigation involved a comparison of the BeBiT-S score with and without B/NHE assistance and demonstrated an increase in BeBiT-S score from a median of 38 points without B/NHE to a median of 60 points during B/NHE use. This underscores the efficacy of the BeBiT-S in capturing and quantifying improvements in bimanual task performance facilitated by B/NHE use. While most participants demonstrated enhanced bimanual task performance with B/NHE assistance, two participants experienced a decline in bimanual task performance. One of them, who experienced a stroke at birth, had extensively adapted to using the impaired hand in activities of daily living, which may explain this lack of improvement. Across all participants, the grasping and stabilizing components showed significant improvements during B/NHE operation (*P* < 0.05 for grasping and stabilizing, Fig. 4C), emphasizing the positive impact of B/NHE-assistance on these essential components of hand function. While the manipulating and lifting components showed improvements, statistical significance was not reached (*P* = 0.102 for manipulating, *P* = 0.619 for lifting). The reaching component, although displaying a marginal decline, did not reach statistical significance (*P* = 0.211). These findings align with the specific design limitations of the hand-exoskeleton device employed in this and other studies [28]. While it can support grasping and stabilizing objects, it does not support proximal movements since it lacks elbow actuators, and may in fact hinder lifting due to added weight. Overall, the findings underscore that the BeBiT-S’ scoring system captures the fundamental aspects of bimanual function, encompassing reaching, grasping, manipulating, and lifting.

However, it is important to acknowledge that assistive devices with different configurations may impact these components in distinct ways. For this reason, development of the BeBiT-S included deliberate efforts to ensure compatibility beyond a single device class. The test was designed to be versatile and applicable to a broad range of assistive technologies, including, for instance, muscle-driven orthoses, functional electrical stimulation (FES), and whole-arm exoskeletons [29]. Even application in assessment of prostheses is conceivable. Nonetheless, further research is needed to confirm compatibility across these different systems.

Another notable observation is the limited representation of mid-range BeBiT-S scores in our sample, with only two participants scoring between 20 and 40 points. This skewed distribution may suggest that the assessment is more sensitive to detecting mild or severe levels of impairment, rather than moderate cases. One possible explanation lies in the structure of the test and its component-based scoring system: participants who are able to perform fundamental elements such as reaching or lifting may quickly accumulate points across multiple components, resulting in a leap from low to high total scores while bypassing the mid-range. As such, the total BeBiT-S score should not be interpreted as a continuous measure of bimanual function.

The BeBiT-S may also prove valuable to assess recovery of bimanual function, e.g., triggered by repeated B/NHE training. Further research is necessary to evaluate whether the BeBiT-S is sensitive to measure improvements throughout the trajectory of such rehabilitation protocols.(4) While the psychometric evaluation of the BeBiT-S has yielded excellent results, it is important to emphasize that these findings require additional validation through larger clinical studies.

## Conclusions

The BeBiT-S assessment proves to be both reliable and valid for evaluating bimanual task performance in stroke survivors, effectively aligning with emerging assistive technologies like B/NHEs. Our study highlights this alignment and demonstrates the BeBiT-S' sensitivity in detecting improvements resulting from innovative assistive technologies in stroke rehabilitation.

## Supplementary Information

Supplementary Material 1.

## Data Availability

No datasets were generated or analysed during the current study.

## Abbreviations

B/NHE: Brain-neural hand exoskeleton
BeBiT-S: Berlin bimanual test for stroke
BCI: Brain-computer interface
BeBiT-T: Berlin bimanual test for tetraplegia
CAHAI: Chedoke arm and hand activity inventory
EEG: Electroencephalography
EOG: Electroocculography
SMR-ERD: Sensori-motor rhythm event-related desynchronization
ICC: Intraclass correlation coefficient
SIS: Stroke impact scale
