# Supplementary material for: The Berlin bimanual test for stroke survivors (BeBiT-S): evaluating exoskeleton-assisted bimanual motor function after stroke
Source: J Neuroeng Rehabil. 2025 Dec 8;22:261. doi: 10.1186/s12984-025-01822-6 (PMC12699850; doi:10.1186/s12984-025-01822-6)
Supplement: Supplementary file 1 — Supplementary Material 1. [file 12984_2025_1822_MOESM1_ESM.pdf]

Berlin Bimanual Test for Stroke (BeBiT-S) Scoring Sheet

Patient´s name/ ID: ___________________________________ Date: ____

Examiner: ____ Comment: _______________________________________

Instructions

Observe the patient during the tasks and check the box if mentioned activity is observable. In case mentioned activity is not observable, the associated box is not checked and no points are given.
Sum up points for each task and each component and transfer score to overview table.

Reaching

Compensatory mechanism = Item is reached by applying compensatory strategies (e.g., hand-over-hand technique, moving bust instead of arm).

Grasping

Compensatory mechanism = Object is grasped with assistance from healthy hand (e.g., inserting the object in the hand by passively flexing the fingers with help of the other hand, using spasticity to hold object).

Manipulating

Slight difficulty = Patient has minor problems to manipulate object successfully.
G*reat difficulty* = Patient has major struggles to manipulate object successfully or applies compensatory strategies (e.g., object held against the trunk, both hands are used to manipulate one item).
If the patient uses only the unaffected hand no points are given.

Lifting

In lifted position = Object are supposed to have no contact to any surface (including trunk) while manipulating. To score, elbows can rest on the table, as long as object is in midair.

1. Open jar

Reaching

Affected arm reaches towards item independently	☐ = 2
− with compensatory mechanism	☐ = 1

Grasping

Affected arm grasps actively	☐ = 2
− with compensatory mechanism	☐ = 1

Stabilization

Affected arm maintains a firm grip throughout task	☐ = 1

Manipulating

Jar is opened without difficulty	☐ = 3
− with slight difficulty	☐ = 2
− with great difficulty	☐ = 1

Lifting

Jar is opened in lifted position	☐ = 2

Total: ___/10

2. Open plastic packet

Reaching

Affected arm reaches towards item independently	☐ = 2
− with compensatory mechanism	☐ = 1

Grasping

Affected arm grasps actively	☐ = 2
− with compensatory mechanism	☐ = 1

Stabilization

Affected arm maintains a firm grip throughout task	☐ = 1

Manipulating

Plastic packet is opened without difficulty	☐ = 5
− with slight difficulty	☐ = 3
− with great difficulty	☐ = 1

Total: ___/10

Berlin Bimanual Test for Stroke (BeBiT-S) Scoring Sheet

Patient´s name/ ID: ___________________________________ Date: ____

Examiner: ____ Comment: _______________________________________

3. Open water bottle

Reaching

Affected arm reaches towards item independently ☐ = 2
- with compensatory mechanism ☐ = 1

Grasping

Affected arm grasps actively ☐ = 2
- with compensatory mechanism ☐ = 1

Stabilization

Affected arm maintains a firm grip throughout task ☐ = 1

Manipulating

Water bottle is opened without difficulty ☐ = 3
- with slight difficulty ☐ = 2
- with great difficulty ☐ = 1

Lifting

Water bottle is opened in lifted position ☐ = 2

Total: ___/10

4. Pour glass of water

Reaching

Affected arm reaches towards item independently ☐ = 2
- with compensatory mechanism ☐ = 1

Grasping

Affected arm grasps actively ☐ = 2
- with compensatory mechanism ☐ = 1

Stabilization

Affected arm maintains a firm grip throughout task ☐ = 1

Manipulating

Glass is poured without difficulty ☐ = 3
- with slight difficulty ☐ = 2
- with great difficulty ☐ = 1

Lifting

Water is poured in lifted position ☐ = 2

Total: ___/10

5. Cut meat-like putty

Reaching

Affected arm reaches towards item independently ☐ = 2
- with compensatory mechanism ☐ = 1

Grasping

Affected hand grasps actively ☐ = 2
- with compensatory mechanism ☐ = 1

Stabilization

Affected arm maintains a firm grip throughout task ☐ = 1

Manipulating

Meat-like putty is cut without difficulty ☐ = 5
- with slight difficulty ☐ = 3
- with great difficulty ☐ = 1

Total: ___/10

6. Clean dish

Reaching

Affected arm reaches towards item independently ☐ = 2
- with compensatory mechanism ☐ = 1

Grasping

Affected arm grasps actively ☐ = 2
- with compensatory mechanism ☐ = 1

Stabilization

Affected arm maintains a firm grip throughout task ☐ = 1

Manipulating

Dish is cleaned without difficulty ☐ = 3
- with slight difficulty ☐ = 2
- with great difficulty ☐ = 1

Lifting

Dish is cleaned in lifted position ☐ = 2

Total = ___/10

7. Lift up pot

Reaching

Affected arm reaches towards item independently ☐ = 2
- with compensatory mechanism ☐ = 1

Grasping

Affected arm grasps actively ☐ = 2
- with compensatory mechanism ☐ = 1

Stabilization

Affected arm maintains a firm grip throughout task ☐ = 1

Lifting

Pot is lifted without difficulty ☐ = 5
- with slight difficulty ☐ = 3
- with great difficulty ☐ = 1

Total = ___/10

8. Open toothpaste tube

Reaching

Affected arm reaches towards item independently ☐ = 2
- with compensatory mechanism ☐ = 1

Grasping

Affected arm grasps actively ☐ = 2
- with compensatory mechanism ☐ = 1

Stabilization

Affected arm maintains a firm grip throughout task ☐ = 1

Manipulating

Toothpaste tube is opened without difficulty ☐ = 3
- with slight difficulty ☐ = 2
- with great difficulty ☐ = 1

Lifting

Toothpaste tube is opened in lifted position ☐ = 2

Total: ___/10

Berlin Bimanual Test for Stroke (BeBiT-S) Scoring Sheet

Patient´s name/ ID: ___________________________________ Date: ____

Examiner: ____ Comment: _______________________________________

9. Apply toothpaste on toothbrush

Reaching

Affected arm reaches towards item independently ☐ = 2

– with compensatory mechanism ☐ = 1

Grasping

Affected arm grasps actively ☐ = 2

– with compensatory mechanism ☐ = 1

Stabilization

Affected arm maintains a firm grip throughout task ☐ = 1

Manipulating

Toothpaste is applied without difficulty ☐ = 3

– with slight difficulty ☐ = 2

– with great difficulty ☐ = 1

Lifting

Toothpaste is applied in lifted position ☐ = 2

Total = ___/10

10. Close zipper of jacket

Reaching

Affected arm reaches towards item independently ☐ = 2

– with compensatory mechanism ☐ = 1

Grasping

Affected arm grasps actively ☐ = 2

– with compensatory mechanism ☐ = 1

Stabilization

Affected arm maintains a firm grip throughout task ☐ = 1

Manipulating

Zipper is closed without difficulty ☐ = 5

– with slight difficulty ☐ = 3

– with great difficulty ☐ = 1

Total = ___/10

**Task**	**Points**
1. Open jar	/10
2. Open plastic packet	/10
3. Open water bottle	/10
4. Pour glass of water	/10
5. Cut meat-like putty	/10
6. Clean dish	/10
7. Lift up pot	/10
8. Open toothpaste tube	/10
9. Apply toothpaste on toothbrush	/10
10. Close zipper of a jacket	/10
**TOTAL**	/100

**Components of hand function**	**Points**
Reaching	/20
Grasping	/20
Stabilizing	/10
Manipulating	/33
Lifting	/17
**TOTAL**	/100
